# 68 The confounder in time of confusion: a case report on Multisystem Inflammatory Syndrome in Children (MIS-C)

**DOI:** 10.1093/rheumatology/keac496.064

**Published:** 2022-10-07

**Authors:** Maureen W Kigara, Musenya E Mwangangi, Angela Migowa

**Affiliations:** Department of Paediatrics and Child Health, Aga Khan University Hospital, Nairobi, Kenya

## Abstract

**Background:**

Multisystem Inflammatory Syndrome in Children (MIS-C) is a novel, life-threatening gghyperinflammatory condition that develops in children about 2–6 weeks^1^ after infection with SARSCoV2. It can create a diagnostic challenge especially if there is no clear history of prior COVID-19 infection or exposure, and because of the varying range of phenotypes and severity. The prognosis however is good as only a 5% mortality has been reported thus far.

**Methods-Case report:**

Feldstein LR, Rose EB, Horwitz SM, Collins JP, Newhams MM, Son MBF, *et al.* Multisystem Inflammatory Syndrome in U.S. Children and Adolescents. N Engl J Med. 2020; 383(4):334–46.

**Case description:**

A 1 year 7-month-old African male presented with persistent fever, vomiting, diarrhoea and poor feeding for 6 days with irritability for 1 day prior to admission. The fever peaked at 40.2C. He had no cough, runny nose or difficulty in breathing. Five days prior to onset of the above symptoms, he was on outpatient treatment for acute tonsillitis and otitis media post exposure to SARS-CoV-2 from the older sibling.

On examination he was noted to have oral thrush.

**68 Table 1:** Summary of initial investigations

WBC (x10^9^/L)	ANC (x10^9^/L)	HB (g/dL)	MCV (fl)	Platelets (10^3^/µL)	BUN (mmol/L)	HCO3 (mmol/L)	Na (mmol/L)	K (mmol/L)	CRP (mg/l)
18.8	11.97	8.3	79	146	15.5	16.9	137	3.49	139

Stool M/C/S: Campylobacter positive Vitamin D: 16.9.

He was admitted to the general paediatric ward and was started intravenous fluids, azithromycin, ceftriaxone, paracetamol, ibuprofen and nystatin. Two days after admission, he was noted to be in fluid refractory shock, that needed inotropic support. He was transferred to Paediatric High Dependency Unit and received Intravenous fluids, oxygen via nasal prongs and transfused with packed red blood cells. On day 3 of admission, he was noted to have red swollen cracked lips, exfoliation of skin, swollen feet with a vasculitic rash and a strawberry tongue. A diagnosis of Kawasaki disease with a differential of multisystem inflammatory syndrome was then made. Further investigations were done

An ECHO was done that showed moderate cardiac dysfunction, with an Ejection Fraction of 51%, dilated chambers, and mild tricuspid and mitral valve regurgitation which was in support of MIS-C according to the CDC case definition.

The child was started on IV immunoglobulin, and subsequent resolution of fevers and hypotension ensued. Three days later, he was noted to have recurrence of fevers and tachypnoea, with persistence of the swelling of the extremities. He received a 2^nd^ dose of IV immunoglobulin and aspirin. Antibiotics were changed to Meropenem, Vancomycin and Fluconazole. His respiratory status worsened, with increased oxygen demand. A chest X-Ray done showed features of pneumonia and a right sided pleural effusion. He was then transferred to ICU. A thoracocentesis was with drainage of 45mls of yellowish blood-stained fluid, with a mixed picture when subjected to Light’s Criteria. At this point, the results from a COVID-19 IgG came back positive, clarifying the diagnosis to be MIS-C. Incidentally, was noted to have hypocalcaemia with low Vitamin D levels.

The patient improved with resolution of shock and the pleural effusions, and was discharged home after 13 days in hospital, on Aspirin, Prednisone, Omeprazole, Calcimax, Aldactone and Furosemide.

He was reviewed as an outpatient 12 days later, and he was clinically stable. A repeat ECHO was done which showed a structurally normal heart with an EF of 70%. He was advised to taper off steroids, stop furosemide and continue aspirin. A subsequent review 1 month later, showed that he was doing well. Aspirin was discontinued and he was discharged from the cardiac clinic. He was to be reviewed again in the rheumatology clinic after 4 months.

**Discussion:**

Our patient’s initial presentation was a confounder as it presented as acute gastroenteritis. His clinical picture evolved to present with features that fulfilled the criteria for Kawasaki disease and the case definitions for Multisystem Inflammatory Syndrome in Children (MIS-C)^1–3^. The initial presentation of gastrointestinal symptoms pointed towards a diagnosis of MIS-C, where gastrointestinal symptoms present in > 80% patients, with some presenting with acute surgical abdomen. His illness progressed rapidly to the point of requiring critical care due to haemodynamic instability, severe cardiac dysfunction and multi-organ involvement (renal, pulmonary, haematological). This is not in keeping with Kawasaki disease, in which <5% of patients develop Kawasaki Disease Shock Syndrome (KDSS) need ICU management. ^1^The child was also noted to have low vitamin D levels. This raises the possibility of a relationship between MIS-C and low vitamin D levels, as Vitamin D is known to be involved in immune modulation.^1,4^

**Conclusion:**

A high index of suspicion of MIS-C is required in children with COVID exposure with otherwise typical presentation of common paediatric emergency ailments. Although the management is symptom-driven, early recognition and institution of management for MIS-C could be important in delaying or avoiding complications. Further studies would be needed to better understand the aetiology of this disease, as well as the disease course, and hence if timely management contributes to a better clinical outcome for our patients.

CDC (2020) Multisystem Inflammatory Syndrome in Children (MIS-C) [Internet]. Centers for Disease Control and Prevention.

^1^ Multisystem inflammatory syndrome in children and adolescents with COVID-19 (2020) Scientific brief:

World Health Organisation.

^2^Royal College of Paediatrics and Child Health (2020) Guidance—Pediatric multisystem inflammatory syndrome temporally associated with COVID-19, 2020.

^3^Kabeerdoss J, Pilania RK, Karkhele R, Kumar TS, Danda D, Singh S. Severe COVID-19, multisystem inflammatory syndrome in children, and Kawasaki disease: immunological mechanisms, clinical manifestations and management. Rheumatol Int. 2021; 41(1):19–32

^4^Yılmaz, K., & Şen, V. (2020). Is vitamin D deficiency a risk factor for COVID-19 in children? Pediatric pulmonology, 55(12), 3595–3601. https://doi.org/10.1002/ppul.25106

